# Multifractal and entropy analysis of resting-state electroencephalography reveals spatial organization in local dynamic functional connectivity

**DOI:** 10.1038/s41598-019-49726-5

**Published:** 2019-09-17

**Authors:** Frigyes Samuel Racz, Orestis Stylianou, Peter Mukli, Andras Eke

**Affiliations:** Semmelweis University, Department of Physiology, 37-47 Tuzolto street, 1094 Budapest, Hungary

**Keywords:** Dynamical systems, Complex networks

## Abstract

Functional connectivity of the brain fluctuates even in resting-state condition. It has been reported recently that fluctuations of global functional network topology and those of individual connections between brain regions expressed multifractal scaling. To expand on these findings, in this study we investigated if multifractality was indeed an inherent property of dynamic functional connectivity (DFC) on the regional level as well. Furthermore, we explored if local DFC showed region-specific differences in its multifractal and entropy-related features. DFC analyses were performed on 62-channel, resting-state electroencephalography recordings of twelve young, healthy subjects. Surrogate data testing verified the true multifractal nature of regional DFC that could be attributed to the presumed nonlinear nature of the underlying processes. Moreover, we found a characteristic spatial distribution of local connectivity dynamics, in that frontal and occipital regions showed stronger long-range correlation and higher degree of multifractality, whereas the highest values of entropy were found over the central and temporal regions. The revealed topology reflected well the underlying resting-state network organization of the brain. The presented results and the proposed analysis framework could improve our understanding on how resting-state brain activity is spatio-temporally organized and may provide potential biomarkers for future clinical research.

## Introduction

Analyzing how different regions of the brain dynamically interact emerged as a new frontier of neuroscience in the past decades^1,2^. Such studies investigating brain functional connectivity (FC) made an immense contribution to the still expanding knowledge on how functional brain networks are organized in space and time^2,3^. Moreover, it has been widely recognized that FC carries great potential for future clinical applications as well^4–6^. Ever since the fluctuating nature of resting-state FC has been explicitly demonstrated^7,8^, a new trend developed within the connectivity field aiming on describing FC in a time-resolved manner^9–11^. This approach investigating dynamic functional connectivity (DFC) allows for a more detailed analysis of brain function either in resting-state condition or during task-modulation^12^. Also, a dynamic approach to FC has been shown by several studies outperforming traditional stationary techniques when it comes to distinguish between healthy and pathological conditions such as schizophrenia^13–16^ or Alzheimer’s disease^17^.

Investigating DFC shed light on several important properties on how neural activity, and most importantly, interactions between neuronal populations are temporally organized. The nonlinear nature of functional coupling between brain regions has been confirmed by Stam *et al*.^18^ based on electroencephalography (EEG) and magnetoencephalography (MEG) recordings. In a functional magnetic resonance imaging (fMRI) study Lahaye and coworkers highlighted the importance of the nonlinear nature of interactions between blood oxygen level dependent (BOLD) signals when identifying significant connections^19^. Their work demonstrated that taking into account the non-linearity of interrelated dynamics makes a model more sensitive than when it is based only on methods of linear correlation. Jia and colleagues^20^ used Sample Entropy^21,22^ (SampEn) analysis for characterizing the temporal complexity of individual connections as well as average FC of each region of interest (ROI), thus stressing the need and importance of revealing non-trivial features of DFC. They created a spatial SampEn map of the brain as well as identified an age-related decrease in the SampEn of connections that link the amygdala to various regions of the cortex; a pattern that was absent in patients with schizophrenia^20^.

DFC has also been shown having several scale-free properties, meaning that fluctuations in neuronal synchronization cannot be characterized by a single time scale. Instead, such processes express long-range correlations (LRC) resulting in a power-law distribution of their statistical properties^23^. Gong and coworkers investigated the dynamic phase synchronization in alpha band EEG between randomly selected brain regions and found that fluctuations in the length of synchronization events followed a scale-invariant pattern^24^. Stam and de Bruin showed that global EEG synchronization also expressed scale-free dynamics in all frequency bands with stronger LRC in eyes closed than eyes open state^25^. Van de Ville and colleagues investigated the dynamics of EEG microstates and revealed that transitions between different microstates also occurred according to scale-free dynamics^26^.

In a recent study utilizing functional near-infrared spectroscopy (fNIRS) imaging and a dynamic graph theoretical approach it has been confirmed that DFC of the prefrontal cortex exhibited not only scale-free but multifractal dynamics^27^, meaning that the scale-free property itself varied over time. In case of multifractality, dynamics has to be characterized with a set of scaling exponents instead of a single scale-free parameter, and the degree of multifractality is captured in the width of the distribution of such scaling exponents^28,29^. The multifractal nature of global DFC was subsequently demonstrated in the whole cortex as well using EEG mapping^30^. Moreover, the scope of observations was extended beyond global FC to synchronization levels between pairs of brain regions, which demonstrated that individual functional connections themselves also fluctuated according to true multifractal dynamics^30^. Finally, a characteristic spatial distribution of the individual connections was found in terms of their multifractal properties, where connections linking regions of the frontal and prefrontal cortex expressed stronger LRC and degree of multifractality^30^. Similarly to previous studies^28,31–33^, it has also been verified that the observed multifractality was – at least partly – a consequence of the nonlinear nature of the investigated process.

From the results of Jia *et al*.^20^ and Racz *et al*.^30^, it is apparent that functional connectivity dynamics vary across different brain regions, which may also bear clinical significance. Interpositioned between global network topology and individual connections, local connectivity of each ROI captures a different aspect of organization; namely of how a given ROI is functionally integrated within the whole dynamic system of the brain. Therefore, the first objective of this study was to verify if multifractality was indeed an inherent property of local DFC. This would enhance our previous approaches where only multifractal properties of global network topology and individual connections were considered^27,30^. In order to realize this goal, we performed DFC analyses on resting-state EEG recordings via estimating dynamic synchronization levels between a set of cortical regions. Subsequently, a measure of local connectivity was computed from the levels of synchronization in a time-resolved manner, yielding time series capturing local DFC for every investigated brain region. Finally, these acquired time series were made subject to multifractal analysis and statistical testing to verify their presumed multifractal nature. Our second aim was to investigate how the scale-free nature of local DFC varied across different ROIs i.e. to see if localization had a significant effect on the multifractal characteristics of local dynamic connectivity. At this end, we also investigated the localization-related differences of another widely used nonlinear measure of signal complexity, Permutation Entropy^34^ (PermEn) in order to offer a more comprehensive description of the spatio-temporal structuring of local DFC. We performed statistical tests to verify if localization had a significant effect on the temporal complexity of DFC, while also performed surrogate data testing to see if the spatial ordering could be destroyed by randomly shuffling electrode localizations. Combining these various methods, our goal was to obtain spatial maps that capture the complexity of local functional connectivity. Acquiring such spatial maps could not only help to enhance our understanding on how the brain as a complex system is spatio-temporally organized, but also provide potential biomarkers for future clinical investigations.

## Results

### Assessing the temporal complexity of local DFC

An openly available dataset containing resting-state, 62-channel EEG recordings of 12, young healthy participants^35^ was analyzed. After preprocessing the raw EEG data (see Materials and Methods), the dynamic i.e. time-dependent synchronization levels between all pairs of channels were estimated using the Synchronization Likelihood (SL) method^36^. This yielded a set of synchronization matrices – one for every time point – describing the dynamically changing connection structure of the cortical functional network. In these, cells contained the actual values of SL between the corresponding ROIs, representing the estimated strength of functional co-operation. Using the synchronization matrices, for every time point we characterized the local functional connectivity of each ROI by its connectivity strength (also termed weighted node degree; for details see Materials and Methods). This analysis eventually produced connectivity strength time series for all ROIs, capturing fluctuations of local DFC. Multifractal properties of these fluctuations were then estimated with focus-based multifractal signal summation conversion^37^ (FMF-SSC). Long-range correlation was described with the monofractal Hurst exponent *H*(2), while the degree of multifractality was captured in the difference between the generalized Hurst exponents obtained at the minimal and maximal generalized moments, termed Δ*H*15. We characterized the temporal complexity of local DFC with the normalized version of PermEn^34^. The whole analysis procedure was carried out in the five frequency bands traditionally used in EEG analysis (delta: 0.5–4 Hz, theta: 4–8 Hz, alpha: 8–13 Hz, beta: 13–30 Hz and gamma: 30–45 Hz).

### Verifying multifractality and nonlinearity in local DFC

When analyzing empirical signals, multifractality can result from several reasons such as a broad power-law distribution of signal values^29^, the presence of different long-range correlations^28^, the nonlinear nature of the process^28^, the finite size of the time series^38^ or mere numerical noise^37^. Since only multifractality related to the presence of LRC and nonlinearity bears physiological significance, we performed surrogate data testing for each investigated time series to exclude the other irrelevant cases of multifractality. In that, (*i*) we checked if the process was indeed scale-free as manifested in a 1/*f*^*β*^ spectra and (*ii*) by shuffling the time series we verified that the scale invariance was indeed a result of LRC. Then (*iii*) by comparing the estimates of multifractality of the actual time series to those of strictly monofractal signals we excluded the possibility of numerical and multifractal background noise. Finally, (*iv*) we performed phase randomization in order to see if the observed multifractality was a consequence of nonlinear dynamics. The presence of nonlinearity was also investigated through PermEn, where (*v*) PermEn of the original time series was compared to those of phase randomized surrogates. Results obtained by this testing framework revealed that local DFC indeed expressed scale-free dynamics, that was found as a consequence of LRC in the signal. The observed multifractality could be clearly distinguished from multifractal background noise and diminished significantly when the nonlinear property of the time series was eliminated by phase randomization. However, PermEn failed to identify nonlinear behavior in the theta, alpha and beta bands. Detailed results for all test steps and frequency bands are shown in Table 1.

**Table 1:** The percentage of the local DFC time series averaged across subjects for all five tests, in all frequency bands (mean ± standard deviation).

	Power-law spectrum	Long-range correlations	Multifractal noise	Nonlinearity (Δ*H*15)	Nonlinearity (PermEn)
Delta	96.9 ± 1.8%	100%	100%	100%	99.6 ± 1.0%
Theta	95.7 ± 2.1%	100%	100%	99.5 ± 1.1%	15.5 ± 6.9%
Alpha	95.6 ± 2.2%	100%	100%	98.7 ± 1.7%	18.4 ± 6.4%
Beta	94.6 ± 2.0%	100%	100%	88.0 ± 7.1%	25.8 ± 14.4%
Gamma	95.3 ± 2.5%	100%	100%	97.0 ± 1.9%	88.3 ± 6.3%

Chi-square test was used separately for each case (frequency band and test) to verify that channel location had no significant effect on the test outcome (*p* > 0.05 in all cases), i.e. the testing framework yielded similar results for all channels.

### Spatial organization in the complexity of local DFC

We used Friedman tests to determine if localization had a significant effect on the assessed values of *H*(2), Δ*H*15 and PermEn. To further explore the existence of a significant topology, Friedman tests were repeated on *n* = 100 spatial surrogates, where for each subject the estimates of *H*(2), Δ*H*15 and PermEn were randomly assigned to the 62 channel locations. To check how similar the spatial distribution of each complexity metric was among individual subjects, we calculated Kendall’s coefficient of concordance^39^ (*W*). We used Cohen’s interpretation guideline^40^, where $W{\epsilon }[0.1\,0.3)$ corresponds to small effect, while $W{\epsilon }[0.3\,0.5)$ and *W* ≥ 0.5 corresponds to moderate and strong effects, respectively. Non-parametric pairwise comparison of all measures in all frequency bands were performed using Kendall’s τ coefficient to see if their topology appeared similar.

In most cases, we found significant regional differences, except for *H*(2) in the delta, and for Δ*H*15 in the theta and alpha bands. Kendall’s *W* mostly indicated small concordance among subjects for *H*(2) and Δ*H*15, however the spatial pattern appeared strongly consistent for PermEn in alpha and higher frequency ranges (shown in detail in Table 2). Spatial surrogate testing further confirmed the existence of a significant topology, as Friedman tests were not significant (*p* > 0.05) in all cases when performed on spatially randomized data. Kendall’s τ revealed that all three parameters showed consistent spatial differences, except when comparing *H*(2) with Δ*H*15 in the delta band, as well as *H*(2) and PermEn with Δ*H*15 in the theta band. These exceptions however are hardly surprising as in these frequency bands the spatial distribution of at least one of the complexity parameters were random/homogenous (see below). When investigating dynamic properties of local FC on the level of intrinsic networks, we found that the revealed channel-wise topology reflected well the functional organization of the brain, especially in the higher frequency bands.

**Table 2:** Results for the Friedman tests (*p*-values) and Kendall’s *W* values for all three investigated measures in all frequency bands.

		Delta	Theta	Alpha	Beta	Gamma
*H*(2)	Friedman *p*	0.1105	**<0.0001**	**<0.0001**	**<0.0001**	**<0.0001**
	Kendall’s *W*	**0.1022**	**0.2748**	**0.2722**	**0.4263**	**0.3203**
Δ*H*15	Friedman *p*	**0.0058**	0.1596	0.5878	**<0.0001**	**<0.0001**
	Kendall’s *W*	**0.1262**	0.0983	0.0791	**0.2164**	**0.2121**
PermEn	Friedman *p*	**0.006**	**0.0001**	**<0.0001**	**<0.0001**	**<0.0001**
	Kendall’s *W*	**0.126**	**0.1499**	**0.5066**	**0.6109**	**0.5886**

Values representing significant effect of localization are marked in bold.

### Spatial maps

**Figure 1:**
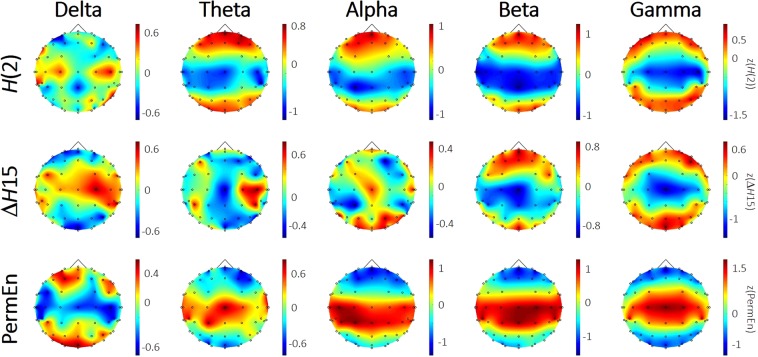
Spatial maps of multifractal parameters and PermEn. Group-averaged spatial maps of *H*(2) (upper), Δ*H*15 (middle) and PermEn (lower) reveal characteristic topology in most of the cases. *H*(2) and PermEn appear to show the opposite pattern to each other, especially in the alpha and higher frequency bands. While the fronto-occipital patterns for the frequency ranges of theta and above are fairly consistent, those of the delta band is clearly seen as distinct. For visualization purposes, data was standardized (z-scored) on the subject-level in each case before group averaging to avoid the influence of inter-subject variability. Note, that standardization has no effect on rank-based statistics.

To observe the spatial distributions of the three investigated dynamic measures, we created group-level spatial maps after standardizing (z-scoring) the values of *H*(2), Δ*H*15 and PermEn on the subject level. Assuming *x* to be be the investigated measure, *z* was obtained as (*x* − *μ*)/*σ*; where *μ* and *σ* are the within-subject mean and standard deviation, respectively. *H*(2) of local DFC was found to express a consistent topology in the theta and higher frequency bands, where frontal-prefrontal and occipital regions could be characterized with higher *H*(2) values, while in the delta band it showed a homogenous distribution (Fig. 1, upper panel). In the delta band, local connectivity dynamics could be characterized with higher Δ*H*15 over the central and temporal regions, however in the beta and gamma bands the opposite topology emerged, with higher values over the frontal-prefrontal and occipital regions (Fig. 1, middle panel). Δ*H*15 was found evenly distributed over the cortex in the theta and alpha bands. When exploring the spatial distribution of PermEn we found that in the delta band the frontal and occipital regions expressed higher temporal complexity. However, in the higher frequency bands again the opposite topology was found, where the central and temporal regions could be described with higher values of PermEn (Fig. 1, lower panel).

### Resting-state networks

**Figure 2:**
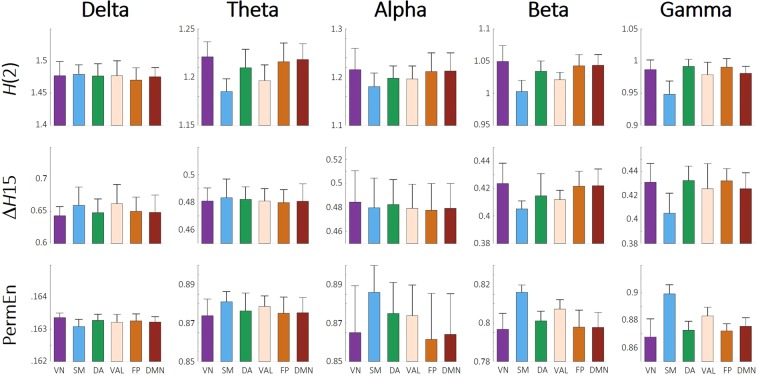
Resting-state network dynamics. Bar plots showing the *H*(2) (upper), ΔH15 (middle) and PermEn (lower) of the 6 RSNs in all five frequency bands. Vertical lines represent standard deviation from the mean. In most cases the somatomotor and ventral attention-limbic networks show clearly distinct characteristics from the rest of the RSNs. VN = visual network; SM = somatomotor; DA = dorsal attention; VAL = ventral attention and limbic; FP = frontoparietal; DM = default mode network.

**Figure 3:**
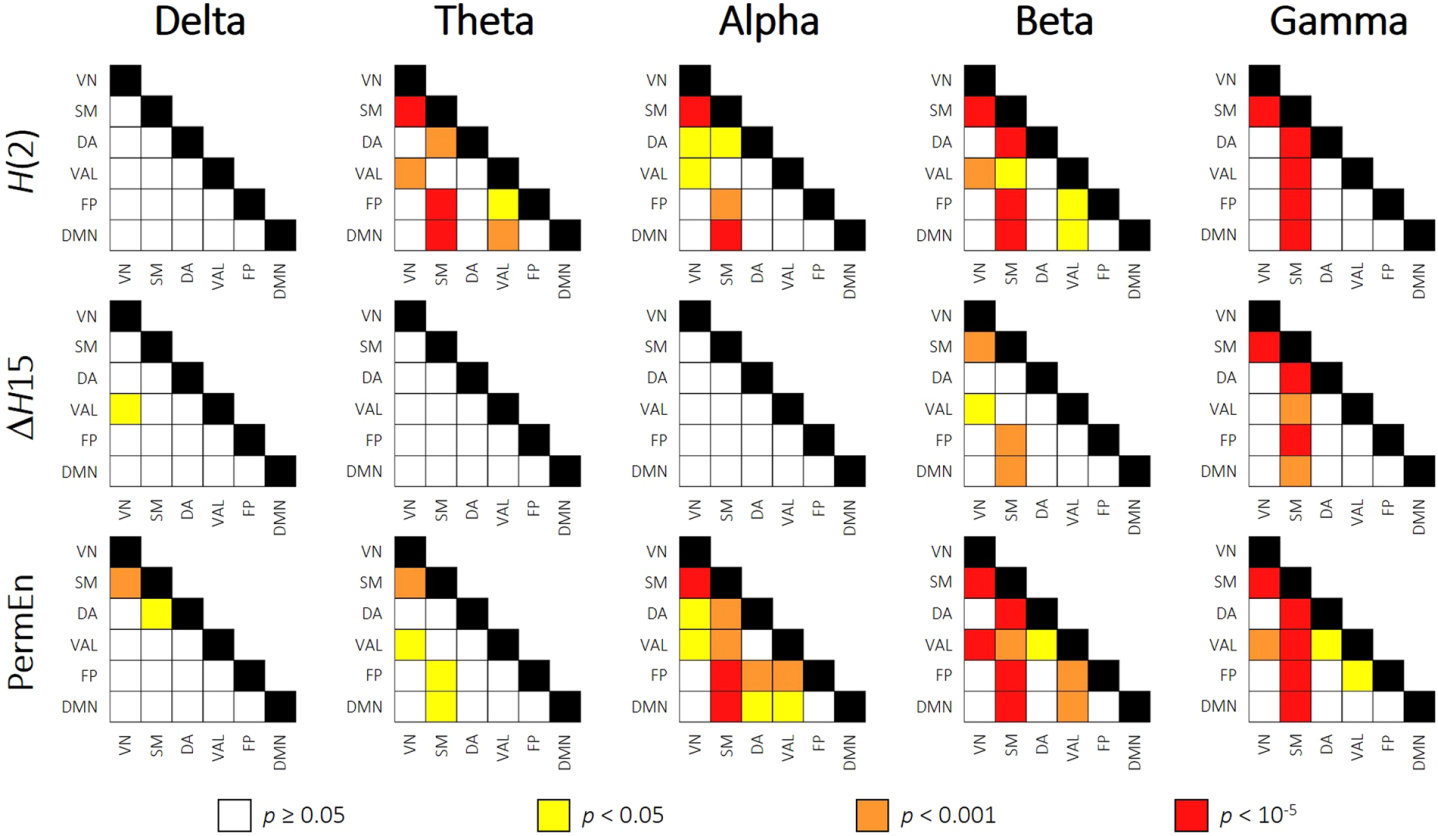
Pairwise comparisons of resting-state networks. Pairwise comparisons of RSN dynamics are presented as matrices, where rows and columns represent the RSNs, while cells contain the result of the pairwise comparison of corresponding RSNs. Different colors mark different levels of significance, while white cells indicate no significant difference as indicated by the legend. Dynamic characteristics of SM separated strongly from the rest of the RSNs in most frequency bands. VN = visual network; SM = somatomotor; DA = dorsal attention; VAL = ventral attention and limbic; FP = frontoparietal; DM = default mode network.

Also, to see if the revealed channel-wise topology resembled the intrinsic network organization of the brain, we sorted the 62 channels into 6 groups to represent the activity of previously established resting-state networks^41^ (RSNs). These included the visual (VN), somatomotor (SM), dorsal attention (DA), ventral attention and limbic (VAL), frontoparietal (FP) and default mode (DMN) networks. Based on the work of Giacometti and colleagues^42^_,_ each EEG channel was assigned to an RSN whose activity the particular electrode most likely recorded (for details, see Materials and Methods and Fig. 7). Following this parcellation, we averaged the *H*(2), Δ*H*15 and PermEn values belonging to the same networks on the subject level. Subsequently, the 6 RSNs were compared with repeated-measures analysis of variance. We found that the previously revealed spatial differences reflected well the intrinsic RSN organization of the brain. Multifractal parameters and PermEn of the RSNs for all frequency bands are presented collectively in Fig. 2, while results of the pairwise comparisons are shown correspondingly in Fig. 3. From the results it is apparent that the visual, frontoparietal and default mode networks expressed mostly similar characteristics. In the higher frequency bands regions with the lowest *H*(2) and Δ*H*15, whilst highest PermEn values were mainly associated with the somatomotor network. Especially in the theta, alpha and gamma bands the combined ventral attention and limbic networks could also be characterized with lower *H*(2) and higher PermEn values than in the visual, frontoparietal and default mode networks. Except for SM in the beta and gamma bands, VAL also expressed lower Δ*H*15 then the rest of RSNs. As expected from the channel-wise spatial maps, RSN dynamics in the delta band showed an opposite pattern when compared to higher frequency bands. Higher Δ*H*15 and lower PermEn values found in SM and VAL attest to this observation. These differences were however less pronounced, as pairwise comparisons proved to be significant only for a limited number of cases (Fig. 3).

## Discussion

In this study, we showed that fluctuations in local DFC expressed complex temporal structuring consistent with a true multifractal model of the observed dynamics. This – similarly to previous studies^27–30^ – was statistically verified by means of surrogate data testing. Further statistical testing revealed that this property indeed manifested independently of brain region, which inferred that multifractality of local DFC was a ubiquitous, genuine property in the whole cortex. Previous studies already demonstrated the scale-free nature of DFC by means of global connectivity^25,26^ as well as on the level of inter-regional connections^24^. The true multifractal nature of both global network topology and that of individual connections were also confirmed recently^27,30^. Our present results contribute to this expanding body of knowledge by demonstrating that multifractality is an inherent property of DFC on the local level as well. Results of surrogate data testing performed on phase randomized surrogates implicate that the observed multifractality is related to the nonlinear nature of regional connectivity dynamics^28,32^. The presence of multifractal dynamics in physiological systems is often interpreted as resulting from stochastic feedback regulation mechanisms^43,44^. This offers a plausible explanation indeed, considering the mostly bidirectional nature of functional connections and the presence of both excitatory and inhibitory synapses in the brain. It is likely that similar regulatory effects could – at least in part – shape the dynamics of regional functional connectivity, however this notion clearly requires further research. Interestingly, the presence of nonlinearity could be more efficiently captured with multifractal time series analysis: phase randomization led to the vanishing of multifractality, that was marked by a prominent decrease in Δ*H*15. On the contrary, PermEn remained almost unaltered following phase randomization instead of increasing, as expected. This suggests that multifractal analysis is a more sensitive technique in identifying nonlinear behavior than PermEn calculation. It should be noted however, that the general applicability of the former is limited as it is a model-based approach, whilst the latter is not^34^.

Although multifractality itself appeared as a universal property of local DFC independent of brain region, we found it manifesting to various extent at different ROIs. More precisely, values of *H*(2) and Δ*H*15 showed characteristic spatial organization in most frequency bands. In the higher range (beta and gamma in particular), the observed topology of *H*(2) and Δ*H*15 appeared quite similar, with higher values over the frontal and occipital regions. This pattern partly resembles the spatial distribution found in the same frequency bands in a previous work of Racz *et al*.^30^ when investigating the multifractal nature of individual connections, where connections with higher *H*(2) and Δ*H*15 values were localized mainly to the frontal and prefrontal cortex. Note however, that in that study the electrode density over the occipital and parietal cortices was sparse, likely preventing this pattern from manifesting fully. The same topology of *H*(2) still turned out to be significant (see Table 2) in the alpha and theta bands, however in the delta band its spatial distribution appeared random. On the contrary, the opposite topology of Δ*H*15 was found in the delta band, showing higher values over dorsal PFC and the temporal lobes. This peculiar behavior of the delta band – i.e. expressing inverse region-specific differences in multifractal characteristics when compared to higher frequency bands – has also been observed previously^30^. While this study understandably limits any further elaboration on the physiological underpinning of these findings, nevertheless it highlights the importance of the observed phenomenon. It appears worthwhile for future research to investigate delta band DFC under more suitable experimental conditions such as during sleep, where delta activity is more prominent. Values of PermEn also showed characteristic regional differences in the theta range and above, that resembled the exact opposite of the topology found in *H*(2) i.e. highest values over the central and temporal regions. In the delta band, a different distribution of PermEn was revealed with lower estimates over the somatomotor and temporal regions, however in that case the state space reconstruction was ill-conditioned: as the embedding dimension allowed for 25! possible permutations, a signal length of order 10^26^ would have been required for a reliable estimate of PermEn. Therefore, these results acquired in the delta band should only be treated as exploratory.

**Figure 4:**
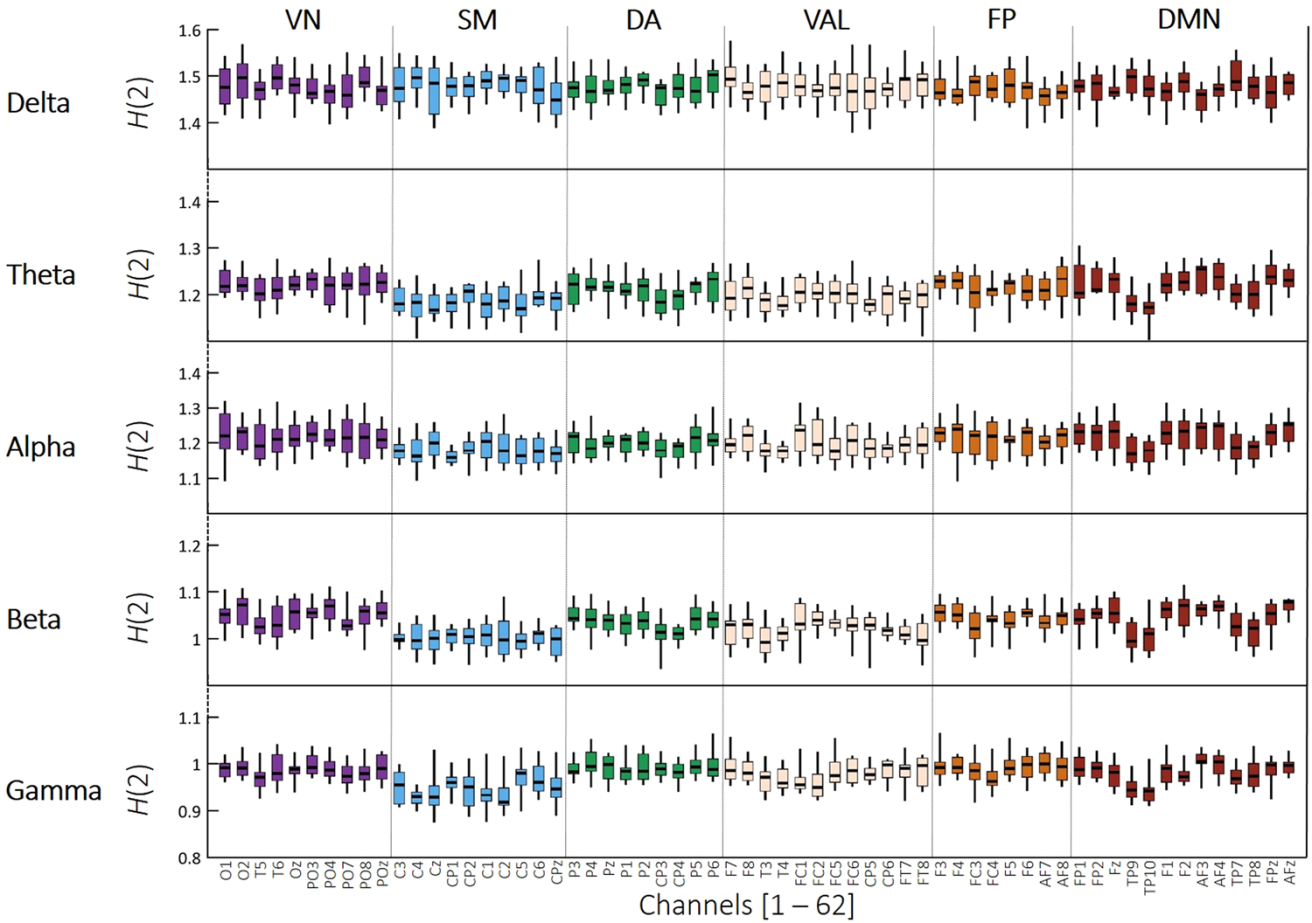
Channel-wise box plots of *H*(2) in all frequency bands. *H*(2) values in the gamma band were found generally in the close vicinity of 1, representing a pure Brownian motion. In the lower frequency bands, *H*(2) mostly fell between 1 and 1.5, indicating an antipersistent process.

*H*(2) values in the gamma band were found in the close vicinity of 1, resembling a pure Brownian random walk (pink noise). In all other frequency bands *H*(2) fell within the range of 1.0 and 1.3 (Fig. 4), characterizing local DFC as an antipersistent process^37^ in consistence with the known nonstationarity of electrophysiological data^23^. This antipersistence implies that an increase in the local functional connectivity of a region is expected to be followed by a subsequent decrease. In other words, a state where the activity of a given ROI becomes more synchronized to those of the rest of the complex system – when the local connectivity of the ROI increases – will likely be followed by a state of functional decoupling.

Interpretation of the results regarding the differences found in Δ*H*15 is more challenging, as the relation of Δ*H*15 to neural activity is not fully established yet. Shimizu *et al*.^45^ reported an increase in the degree of multifractality in the occipital cortex during visual stimulation when investigating brain function through the BOLD signal. In a different fMRI study Ciuciu *et al*.^46^ utilized an auditory response paradigm and observed an increase in the degree of multifractality in the cerebellum, basal ganglia and fronto-parietal regions, while a decrease in the auditory and attentional systems. The cognitive relevance of multifractality was also investigated by Wink *et al*.^47^, finding that increased width of the multifractal spectrum (i.e. stronger multifractality) in the inferior frontal cortex was associated with faster response times in a face recognition task, but also with weaker accuracy. Δ*H*15 of local DFC reflects on the range in which scaling of local DFC varies over time, that also corresponds to the strength of nonlinearity in the process. From our results it appears that resting-state dynamic connectivity in the frontal and prefrontal regions (and also in the visual cortex) is regulated through more enhanced feedback mechanisms, giving rise to increased variability in the scaling of local DFC. An opposite explanation seems reasonable regarding the somatomotor cortex, leastwise in resting-state condition. This behavior is hardly surprising though, as during rest the somatomotor system is presumably idle. Finally, the spatial pattern revealed for PermEn implicates a higher level of complexity in local DFC of the somatomotor system. From a different perspective, where white noise is considered as an infinitely complex process and therefore can be characterized with maximal entropy (in the normalized case, 1), local FC dynamics in the somatomotor system can be regarded closer to a random process when compared to the rest of the brain. This is well reflected in the values of PermEn usually falling between 0.8 and 0.9, meaning that local DFC is a highly complex process, although clearly distinguishable from white noise. It should be reiterated, that in most frequency bands the topology of PermEn highly resembled – although inversely – that of *H*(2). This level of resemblance raises questions about PermEn as a complexity estimator, which could be affected by the long-range correlations in the process. A recent work of Xiong and colleagues^48^ thoroughly investigated – beside other effects – the influence of LRCs on the performance of entropy estimators. According to their results, kernel-type estimators (such as PermEn) are biased by overestimating the absolute (theoretical) value of entropy, however this bias remains constant and very stable, independently of the strength of LRC present in the signal^48^. Thus, even though PermEn would not necessarily yield the correct values of absolute entropy, the differences observed between various regions indeed indicate different levels of complexity, and not just merely reflect the different levels of long-range correlation. The relevance of this in this study is with PermEn appearing the most sensitive of the three parameters investigated, identifying the strongest channelwise spatial concordance with Kendall’s *W* > 0.5 in the alpha, beta and gamma bands.

By grouping the EEG channels to resemble intrinsic resting-state networks of the brain, we found that locations with markedly different dynamical properties indeed corresponded well to intrinsic functional networks. Most prominently, it was revealed that those regions with the lowest *H*(2), Δ*H*15 and highest PermEn values in most frequency bands corresponded mainly to the somatomotor network, the dorsal attention network and the combined ventral attention and limbic networks. Conversely, the highest *H*(2) values were consistently found in the visual, frontoparietal and default mode networks. Similar differences between scaling properties of resting-state networks were also observed in previous studies^49,50^. Specifically, He^50^ investigated the scale-free nature of the BOLD signal in numerous brain regions using detrended fluctuation analysis. Significant differences were identified between the monofractal Hurst exponent of various RSNs. In particular, the highest values were found in the default and visual networks, lowest values were produced by the motor network, the thalamus, the hippocampus and the cerebellum; while Hurst exponents of the attention and saliency networks were found in between^50^. Although our results were acquired using a different imaging modality, they are remarkably consistent with the results presented by He^50^, expanding on those by showing that not only monofractal, but multifractal and entropy-based measures, too, show significant differences among various RSN dynamics.

Several studies investigated the correspondence between RNSs reconstructed using fMRI and narrow-band electrophysiological activity in order to identify relationships between various RNSs and specific frequency bands. Some patterns were consistently verified by these studies, however there is also a considerable amount of inconsistency among the results. The visual network was found to show generally good correspondence to electrophysiological activity in all frequency bands except gamma^51^, with the correlation being the highest with the alpha and beta bands^51,52^. The somatomotor network was frequently reported to be strongly related to beta band activity^51,53^ and connections among the SM network could be best explained by beta band MEG activity^52^. Brain function within areas of the dorsal attention network was also most strongly related to alpha and beta band oscillations^51,54^. The electrophysiological correlates of the frontoparietal network were found mainly in the theta and gamma bands^52,54^. Finally, findings in the literature are the most inconsistent regarding the default mode network, which was reported to be associated with generally all frequency bands, albeit to a different extent^51,53,54^. Although to some degree most studies managed to associate RSNs to different frequency bands, a general conclusion was often drawn that RSNs most likely emerge from neural activity involving multiple frequencies. This was first demonstrated by Mantini *et al*.^51^, who showed that the dynamics of six investigated RSNs could be associated to various extent with electrophysiological oscillations of more than one frequency range. In fact, each RSN could be characterized by a specific set of electrophysiological correlates including all five frequency bands^51^. Similar conclusions were drawn by Hipp *et al*.^53^, who showed that even though there were cases where the activity of an RSN strongly represented neuronal activity in a specific frequency band (SM and beta), in general RSNs were more likely to be associated with a mixture of concurrent activities at multiple frequencies. Note, that VN, SM and DA (among others such as the auditory network) are considered as parts of the extrinsic, while FP and DMN as constituents of the intrinsic higher order systems of the brain^54,55^ and thus they are often expected to show similar inter-system and different between-system characteristics. In this present study, generally similar differences were found in the dynamics of local FC on the RSN level in all frequency bands. The SM and VAL networks were found most of the time to express different dynamic characteristics than the rest of RSNs (see Fig. 2), therefore our findings do not resemble the extrinsic-intrinsic organization of the brain. However in Fig. 3 it is apparent that each frequency band could be characterized with a unique set of differences, therefore our results are in support of those suggesting that RSNs are more likely to be associated with a mixture of neural activities emerging from a broad range of frequencies^51,53^.

Finally, we have to address certain limitations of this study. Although we were able to confirm the true multifractal nature of local resting-state DFC, the analyzed dataset consisted only of measurements with eyes closed. In fact, global EEG dynamics^56^ and also scale-free properties of global DFC^25,30^ show several differences between eyes open and eyes closed states. Hence, in order to assess the influence of cortical desynchronization on multifractal properties of local DFC it would be indeed instructive to investigate state-related differences. Alterations in functional connectivity related to various task-response paradigms were already demonstrated using either static^57–59^ or dynamic^12,60^ approaches alike. As multifractal analysis proved to be capable of identifying cortical regions with increased stimulation^45,46^, its combination with DFC and entropy analysis could provide an even more sensitive tool in investigating the neuronal response to various external stimuli. Also, for the sake of simplicity in this study we characterized local functional connectivity only with one measure that is basically equivalent to the weighted node degree^61^. However, several other local properties of functional networks can be considered, such as the clustering coefficient^62^, local efficiency^63^ or centrality-related measures^64^. Inclusion of such measures in future studies could be beneficial. Probably the most severe drawback of the present study is the lack of exact source localization of the EEG signal, that would allow for a more precise brain parcellation and more direct estimation of neural activity of various RSNs^65,66^. In this regard, our procedure to parcellate the set of EEG electrodes in reference to intrinsic RNSs based on the probability maps provided by Giacometti *et al*.^42^ (see Materials and Methods) can be only regarded as a crude approximation, yet capable of producing results comparable to previous reports in the literature. The reason why we decided to follow this approach was that we would have liked to propose an analysis pipeline that was fully automatized and relied on EEG data only. For the same reason, all EEG preprocessing was carried out using automated algorithms. Likewise, starting parameters of the analysis tools used during the analysis (i.e. SL, FMF-SSC and PermEn) were defined according to objective, purely data-driven criteria. The motivation behind these considerations was that this would allow such pipeline to be implemented with ease in a broad range of scenarios in possible future studies, including clinical conditions as well. Finally, it would be instructional to see how the results may depend on the electrode density of the measuring equipment. In order to address this issue, we repeated all steps of the analysis procedure using data only from the 19 electrodes localized at the international 10–20 positions. Detailed results of this approach are attached as Supplementary Material. We found that the characteristic topologies revealed based on all 62 channels could be very well reproduced using only 19 channels covering the whole cortex (see Fig. S1). Results regarding RSN dynamics (Figs S2 and S3**)** and concordance among subjects (Table S1) were also comparable, implicating that the patterns presented in this study were generally independent of electrode density.

## Conclusions

In this study, we demonstrated that DFC expressed multifractal dynamics when investigated on the local level and this property could be thoroughly verified through surrogate data testing. On this ground, we were able to characterize the temporal complexity of local DFC through its multifractal properties, utilizing also Permutation Entropy to have a more comprehensive description of system dynamics. We found that temporal characteristics of DFC varied significantly over the cortex, yielding characteristic spatial distributions of multifractal and entropy-related features. Moreover, the observed topologies reflected well the underlying functional organization of the brain. Our results shed light on previously unobserved features of spatio-temporal brain dynamics that are not only meaningful for a better understanding of physiological brain function, but could carry potential in future clinical applications as well.

## Materials and Methods

### Data and participants

In this study we analyzed a publicly available dataset of resting-state EEG recordings published and made freely available by Sockeel and colleagues^35^. The dataset consisted of the EEG recordings of twelve young, healthy participants (age 26.6 ± 2.1 years, 6 female, all right-handed). Measurements were carried out using a 62-channel BrainAmp system with a sampling rate of 5 kHz. The reference electrode was localized in the Cz position while a ground electrode was placed below the Oz position. During the measurements subjects were lying supine with their eyes closed while a recording equivalent to the sound produced by an MRI system was played to mimic the conditions of an MRI measurement. Similarly to Sockeel *et al*.^35^, only the last 300 seconds of the datasets (containing the resting-state recordings with eyes closed) were used in our study. Data was visually inspected and artifact-free segments with a length of 2^18^ data points were selected for further analysis. The original study of Sockeel *et al*.^35^ was approved by the local ethics committee (Comité de Protection des Personnes–Ile-de-France under the number CPP DGS2007-0555), measurements were carried out in accordance with the Declaration of Helsinki and all participants provided written informed consent.

### Preprocessing

Preprocessing of raw EEG data was performed in Matlab (The Mathworks, Natick, MA, USA) using the Batch Electroencephalography Automated Preprocessing Platform^67^ (BEAPP). BEAPP operates using custom functions and scripts along with methods of the EEGLAB software package^68^. Data preprocessing consisted of the following steps of the BEAPP pipeline: (*i*) Raw data was first band-pass filtered with lower and upper cutoff frequencies 0.5 Hz and 250 Hz, respectively, with additional ‘cleanline’ filtering to remove line noise at 50 Hz. (*ii*) Band-pass filtered data was then downsampled from 5 kHz to 500 Hz. Subsequently, (*iii*) artifact removal was carried out automatically using the Harvard Automated Processing Pipeline for Electroencephalography^69^ (HAPPE). HAPPE includes multiple steps of denoising such as a wavelet-enhanced independent component analysis (W-ICA) approach^69^ followed by independent component analysis (ICA) with automated component rejection using the Multiple Artifact Rejection Algorithm^70,71^ (MARA). Finally, (*iv*) data was again band-pass filtered to the frequency bands traditionally used in EEG analysis (delta: 0.5–4 Hz, theta: 4–8 Hz, alpha: 8–13 Hz, beta: 13–30 Hz and gamma: 30–45 Hz) using a 5th order Butterworth filter.

### Dynamic functional connectivity estimation

Functional connectivity between all possible pairs of brain regions was estimated using the Synchronization Likelihood (SL) method^36^. SL utilizes a temporal embedding-based state-space reconstruction approach^72^ of dynamic processes *x*(*t*) = *x*_1_, *x*_2_, … *x*_*T*_ and *y*(*t*) = *y*_1_, *y*_2_, … *y*_*T*_ and estimates the probability of their generalized synchronization^73^. First, *x*(*t*) and similarly, *y*(*t*) are reconstructed in an *m* dimensional state space as a set of state space vectors using temporal embedding^72^ with time lag *L* as

1 $$X(t)=[{x}_{t},\,{x}_{t-L},\,\ldots \,{x}_{t-(m-1)L}],Y(t)=[{y}_{t},\,{y}_{t-L},\ldots \,{y}_{t-(m-1)L}].$$

Then, for each time point *t*, the *k* nearest neighbors of *X*(*t*) and *Y*(*t*) within a local neighborhood are identified by their Euclidean distance. Finally, SL for every time point *t* is calculated as the conditional probability that the nearest neighbors of *X*(*t*) can be found at the same time points as the nearest neighbors of *Y*(*t*). Parameters of the SL algorithm (mainly *m* and *L*) were set in each case to fit the frequency bands and data sampling rate according to Montez *et al*.^74^ and are shown in Table 3. State space vectors can be close to each other not only due to recurrence but simply because they are close in time^75^. Therefore, to diminish this effect of temporal autocorrelation the direct neighborhood – as defined by the parameter *w*_1_ – of the actual state space vector *X*(*t*) is always excluded from the nearest neighbor search. Finally, the parameter *w*_2_ (w_2_ ≫ w_1_) defines the fixed neighborhood of *X*(t) where the nearest neighbor search is performed. For a detailed description and evaluation of the method the reader is referred to the original paper of Stam and van Dijk^36^. With SL being a probability-type measure it takes on values between 0 and 1 representing independence and complete synchronization, respectively. SL has several properties that make it an appealing choice for EEG-based DFC analysis as it is able to identify nonlinear dependencies between processes, robust against non-stationarities and it works in a time-resolved manner^25,36^. We set the internal thresholding parameter (that basically defines the number of nearest neighbors searched) to 0.05 as in previous studies^25,30,76^.

**Table 3:** Starting parameters of Synchronization Likelihood for all frequency bands.

	*m*	*L*	*w*_1_	*w*_2_
Delta	25	41	1968	2967
Theta	7	20	240	1239
Alpha	6	12	120	1119
Beta	8	5	70	1069
Gamma	6	3	30	1029

A method on how to objectively define *m*, *L*, *w*_1_ and *w*_2_ on data with explicit frequency limits is presented in Montez *et al*.^74^. The same values of *m* and *L* were used for Permutation Entropy calculation in each frequency band.

**Figure 5:**
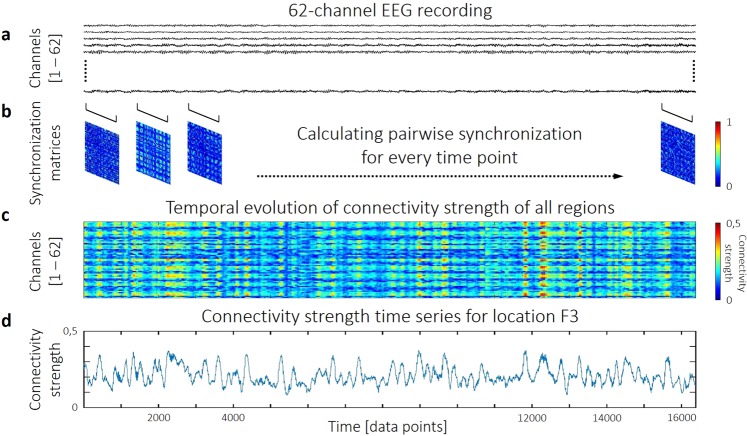
The process of dynamic functional connectivity estimation. Pairwise Synchronization Likelihood estimation is performed on the 62-channel EEG data (**a**) in a time-resolved manner. This yields a synchronization matrix for every time point (**b**). From these matrices, connectivity strength of each region can be calculated in each instance (**c**), yielding connectivity strength time series. As a demonstration, the connectivity strength time series of region F3 is shown (**d**) from a representative subject, calculated from alpha band activity.

After calculating the pairwise SL time series from the 62-channel preprocessed EEG data (Fig. 5a), the acquired results could be organized into a set of synchronization matrices. More precisely, each matrix described the functional connection structure of the cortex at time point *t* (Fig. 5b). Based on these, local functional connectivity of each region was characterized with the connectivity strength (*S*) for every time point (Fig. 5c). *S* of channel *i* was calculated according to

2 $${S}_{i}(t)=\frac{1}{N-1}\mathop{\sum }\limits_{j=1,\,j\ne i}^{N}\,S{L}_{ij}(t)$$

where *SL*_*ij*_(*t*) is the value of SL between channels *i* and *j* at time poin*t t* and *N* is the number of channels. In a network theoretical approach the connectivity strength of a node is equivalent to its normalized weighted node degree^61^ and thus it captures the connectedness of the given ROI to the rest of the functional network. Finally, this procedure resulted in a connectivity strength time series for every channel (Fig. 5d), that captured the dynamics of local functional connectivity of the according brain region.

### Multifractal time series analysis

**Figure 6:**
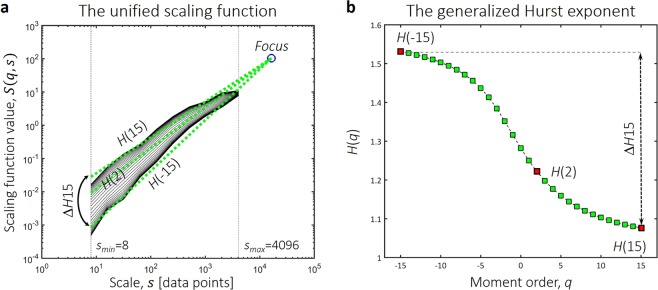
End-point parameters of multifractal time series analysis. The scaling function (**a**) is acquired by multifractal signal summation conversion. In that, standard deviation is calculated as a function of scale and the process is repeated along several statistical moments. The generalized (*q*-dependent) Hurst exponent (**b**) is acquired via linear regression with the focus used as a reference point. Global scale-invariance is described by *H*(2), while the degree of multifractality is captured as the difference between *H*(*q*) calculated at the minimal (−15) and maximal (15) moments.

The first 2^14^ data points of the connectivity strength time series were subjected to FMF-SSC analysis^37^ to estimate their multifractal characteristics. The general (monofractal) signal summation method^77^ (SSC) consists of the following steps: (*i*) the data is divided into segments (windows) of size *s*, (*ii*) for each segment the local linear trend is removed to avoid effects of non-stationarity, then (*iii*) the standard deviation σ of the detrended data is calculated in every window and finally (*iv*) averaged along the windows. Subsequently v) the procedure described in steps (*i*–*iv*) is carried out for different scales (windows of different size) ranging from *s*_*min*_ to *s*_*max*_. This yields a scaling function *S*(*s*) that is in general the averaged σ calculated as a function of window size. In case of scale-free signals, a power-law function can be fitted to *S*(*s*). The scaling exponent obtained this way is the monofractal Hurst exponent *H*, capturing the long-range correlation property of the process^23^. In the multifractal extension of SSC, the procedure described in steps (*i*–*v*) is repeated using a set of generalized moments (of order *q*) to magnify the effect of small (negative *q* values) and large (positive *q* values) fluctuations. This sequence of calculation yields the unified scaling function^37^ *S*(*q*, *s*) (Fig. 6a) according to

3 $$S(q,s)={\{\frac{1}{{N}_{s}}\mathop{\sum }\limits_{\upsilon =1}^{{N}_{s}}\sigma {(\upsilon ,s)}^{q}\}}^{\frac{1}{q}}$$

where *N*_*s*_ is the number of windows at scale *s* and *υ* is the index of the actual window of size *s*. Apart from calculating standard deviation instead of fluctuation as the appropriate statistical measure, signal summation conversion is basically equivalent to the more widely used detrended fluctuation analysis^78,79^. Note, that in case of finite empirical time series, replacing *s* in (3) with the total signal length basically eliminates the sum from the formula, thus making it independent from *q*. This results in the convergence of the values of *S*(*q*, *s*) calculated at different *q*s into a single point termed the *focus*^37^. Therefore, the focus can be used as a reference point during linear regression, in that power-law functions are fitted to *S*(*q*, *s*) at each *q* (Fig. 6a). This procedure yields the generalized Hurst exponent, *H*(*q*) (Fig. 6b) that is defined as the scaling exponent of the fitted power-law function at generalized moment *q*. When the scaling function is plotted using double logarithmic scaling as in Fig. 6a, the fitted power-law functions appear as linear and estimates of *H*(*q*) are obtained as the slope of the function fitted to *S*(*q*, *s*) at moment *q*. Apart from making multifractal analysis robust in case of finite empirical signals, the focus-based regression also helps in enforcing the monotonously decreasing property of *H*(*q*)^37^, i.e. *H*(*q*_1_) ≥ *H*(*q*_2_) if *q*_1_ < *q*_2_. The step-by-step process of FMF-SSC analysis is also illustrated in the Supplementary Video.

Parameters of the FMF-SSC analysis were set according to Mukli *et al*.^37^, with *s*_*min*_ = 2^3^, *s*_*max*_ = 2^12^ and $q{\epsilon }\{-15,-\,14,\ldots ,+\,14,+\,15\}$. Multifractal properties were captured in two end-point parameters (Fig. 6b): *H*(2) which is equivalent to the monofractal Hurst exponent reflecting on the global long-range correlations in the signal^23^, and Δ*H*15 that is the difference between *H*(−15) and *H*(15), capturing the degree of multifractality in the process^37^. It is important to point out that these two measures adequately represent the multifractal nature of a process equivalently to the widely used singularity (also often termed multifractal) spectrum, as the latter can be obtained from the generalized Hurst exponent via Legendre transformation^29,37^. Consequently, *H*(2) is analogous – although not strictly equivalent – to the centre of the singularity spectrum, while Δ*H*15 captures the same property as the width of the singularity spectrum^37^.

### Permutation entropy

As a first step of PermEn calculation, the investigated time series *x*(*t*) = *x*_1_, *x*_2_, … *x*_*T*_ is converted into a set of *m*-dimensional vectors *X*(*t*) using temporal embedding^72^ with time lag *L*. Then, values in each vector are replaced by their ranks within the actual vector. This step basically converts each vector into one of *m*! possible permutations of order *m*. The relative frequency of each permutation of type π in the process can be then calculated as

4 $$p(\pi )=\frac{\#\,of\,type\,\pi \,permutation}{T-(m-1)L+1}$$

giving the best estimate on the frequency of π in a finite time series^34^. Finally, Bandt and Pompe^34^ defined permutation entropy of order *m* as

5 $$E(m)=-\,\sum p(\pi )\log \,p(\pi )$$

with the sum running over all *m*! possible π permutations of order *m*. Also, the upper limit of *E*(*m*) always appear to be equal to $\log \,m!$, therefore its normalized form can be acquired by dividing it by this upper limit. Thus 0 ≤ *E*(*m*) ≤ 1 typically corresponds to different levels of complexity, with 0 standing for a completely predictable i.e. minimally complex, while 1 a maximally unpredictable i.e. random process^34^. Although there is no theoretical limit regarding the order *m*, by practical and computational considerations *m* is usually chosen in the range $m{\epsilon }\{3\ldots 7\}$, however with sufficient time series length higher values can be applied as well^34^. Also, in many applications *L* = 1 is chosen, however in signals with explicit frequency limits it is advised to choose *m* and *L* so that the fluctuations present in the signal are well represented by the embedding^80,81^. Therefore, to remain consistent within our analyses, for all frequency bands we chose the same values of *m* and *L* as when calculating SL, as listed in Table 3.

### Surrogate data testing

For the testing of power-law spectra, surrogate time series with equal length and spectral slope were generated using the spectral synthesis method^82^. The spectral slope *β* of the DFC time series was estimated by fitting a power-law function on their power spectrum using linear regression. Goodness of fit of the power-law function was assessed by the Kolmogorov distance *D* according to Clauset *et al*.^83^. If *D* of the original time series was within the mean ± 2σ range of those acquired from surrogate data, the time series was considered having a power-law spectrum^50,83^. For each time series 40 surrogate signals were generated, as this number corresponds to a 95% and 97.5% confidence level for two-sided and one-sided tests, respectively^84,85^.

Surrogates for LRC testing were created by shuffling the original time series, as shuffling destroys all long-range correlations and reduces the process into pure white noise, while having no effect on the distribution of the values^28,29^. As *H*(2) of the original DFC time series were found always above 0.8, if it was outside the mean + 2σ range of those acquired from the surrogate time series the presence of LRC was verified. Again, 40 surrogates were generated for each time series.

Monofractal time series (*n* = 40) with equal length and *H*(2) were generated using the method of Davies and Harte^86^. Δ*H*15 values were acquired with FMF-SSC, and if Δ*H*15 of the DFC time series was larger than the mean + 2σ acquired from the monofractal signals, the case of multifractal background noise could be excluded.

Surrogates for the testing of nonlinearity (*n* = 40) were generated by Fourier transforming the original signal, randomizing its phases and then performing inverse Fourier transformation^84^. If the Δ*H*15 of the DFC time series was larger than the mean + 2σ acquired from the surrogates, the observed multifractality was considered as a consequence of nonlinearity. PermEn of the surrogates were also calculated and compared to those of DFC time series. If PermEn of the original time series was outside the mean ± 2σ range acquired from surrogates, the presence of nonlinearity was verified.

### Brain parcellations

**Figure 7:**
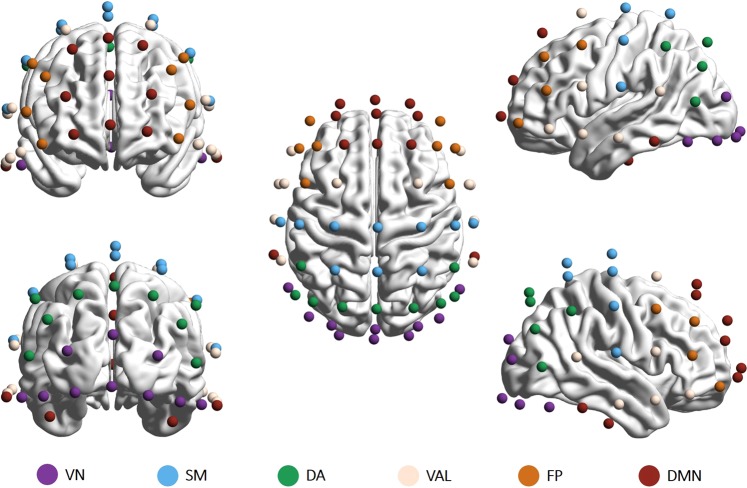
Electrodes were grouped to represent six resting-state networks: the visual network (VN, 10 channels), the somatomotor network (SM, 10 channels), the dorsal attention network (DA, 9 channels), the combined ventral attention and limbic networks (VAL, 12 channels), the frontoparietal network (FP, 8 channels) and the default mode network (DMN, 13 channels). Brain maps were created using the BrainNet Viewer software^87^ after electrode positions were transformed to match a template head using SPM 12b^88^. VN = visual network; SM = somatomotor; DA = dorsal attention; VAL = ventral attention and limbic; FP = frontoparietal; DM = default mode network.

Brain parcellation was carried out manually according to the probability maps reported by Giacometti and colleagues^42^. These maps contain probabilities for EEG electrodes positioned in the 10–20, 10–10 and 10–5 configurations on which anatomical or functional network they most likely monitor^42^. We applied a non-overlapping parcellation scheme presented in Giacometti *et al*.^42^ where EEG electrodes were grouped to represent the activity of 7 intrinsic functional networks as identified by Yeo *et al*.^41^. Since some EEG electrodes were found to register the simultaneous activity of more than one intrinsic network^42^, in such cases the electrode was always assigned to the intrinsic network with the highest probability to achieve a non-overlapping parcellation. Also, as the electrode positions monitoring the ventral attention and limbic networks were strongly overlapping, we decided to treat these two networks jointly, resulting in a final number of 6 networks and their corresponding groups of channels (Fig. 7). Finally, the *H*(2), Δ*H*15 and PermEn values were averaged within each network on the subject level.

### Statistical analysis

As requirements of a repeated measures analysis of variance were not fulfilled – because of the non-normal distribution of values in at least one of the channels (Kolmogorov-Smirnov test, *p* < 0.05) – we performed Friedman tests with a significance level of α_s_ = 0.05 to investigate the effect of channel localization on the values of *H*(2), Δ*H*15 and PermEn. To further verify the existence of a significant topological distribution, Friedman tests were also performed on *n* = 100 spatial surrogates, where for each subject the estimated values of *H*(2), Δ*H*15 and PermEn were assigned to the 62 channel locations randomly. The accordance in the ranking of different electrode positions among subjects was estimated using Kendall’s coefficient of concordance^39^. Kendall’s *W* captures the similarity in the rank order of values and thus it reflects how similar the topological distribution appeared among the subjects. Non-parametric assessment of pair-wise correlation between the ranks of different complexity parameters was carried out using Kendall’s τ coefficient to determine whether the same subjects were responsible for the observed within-subject significant differences. When evaluating the difference between the dynamical measures of the 6 intrinsic brain networks, repeated measures analysis of variance could be applied as the datasets were normally distributed (Kolmogorov-Smirnov test, *p* > 0.05 in all cases) and the assumption of sphericity was not violated (Mauchley’s test, *p* > 0.05). Pairwise multiple comparisons between the networks were carried out separately for values of *H*(2), Δ*H*15 and PermEn using Bonferroni post-hoc tests with a significance level of α_s_ = 0.05. Statistical analyses were performed using StatSoft Statistica 13.2.

## Supplementary information

Supplementary Video

Supplementary Material

## Data Availability

The EEG data analyzed in this study was made publicly available without restrictions by Sockeel, *et al*.^35^ at http://megfront.meg.chups.jussieu.fr/MEEG_data/data.tgz and http://datadryad.org/review?doi=doi:10.5061/dryad.v9f16.
