# Supplementary material for: Multifractal and entropy analysis of resting-state electroencephalography reveals spatial organization in local dynamic functional connectivity
Source: Sci Rep. 2019 Sep 17;9:13474. doi: 10.1038/s41598-019-49726-5 (PMC6748940; doi:10.1038/s41598-019-49726-5)
Supplement: Supplementary file 2 — Supplementary Material [file 41598_2019_49726_MOESM2_ESM.pdf]

Multifractal and entropy analysis of resting-state electroencephalography reveals spatial organization in local dynamic functional connectivity – Supplementary Material

Authors: Frigyes Samuel Racz[1], Orestis Stylianou[1], Peter Mukli[1] and Andras Eke[1,*]

[1]Semmelweis University, Department of Physiology, 37-47 Tuzolto street, 1094 Budapest, Hungary

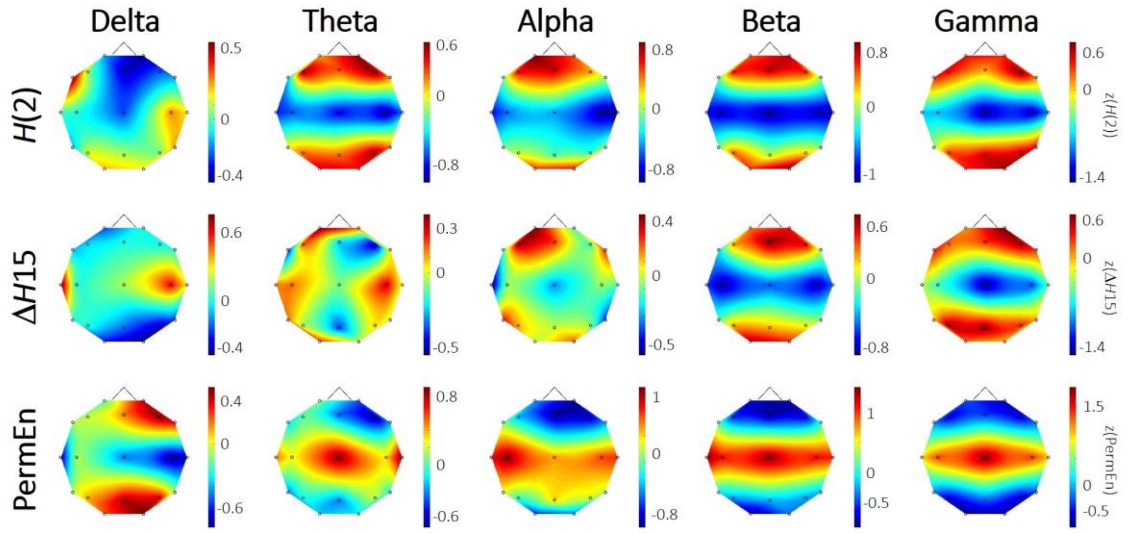

Figure S1. Spatial maps using only 19 electrodes of the 10-20 system. As previously, the group-averaged spatial maps were generated following standardization of the values on the subject level. $H(2)$ were found the highest over the frontal and occipital regions in frequency ranges except delta. This same spatial distribution characterized $\Delta H15$ in the beta and gamma bands, while the opposite topology – highest values over central and temporal regions – was revealed for PermEn in the theta, alpha, beta and gamma bands. Thus, in all cases where spatial differences were significant, the observed topologies closely resembled those found when using data from all 62-channels.

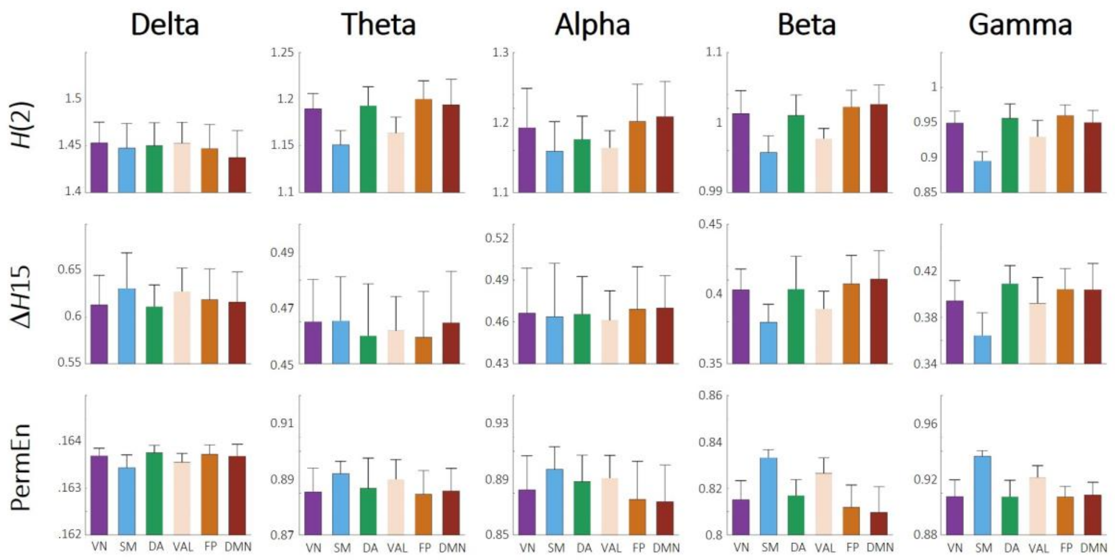

Figure S2. Resting-state network dynamics based on the 19 electrodes of the 10-20 system. Again, the SM and VAL networks could be characterized with lower $H(2)$ and $\Delta H15$ values than the rest of the RSNs, especially in higher frequency bands. On the contrary, the SM and VAL networks produced higher PermEn values in the same range. This results are also well in line with those found by using all 62 channels. VN = visual network; SM = somatomotor; DA = dorsal attention; VAL = ventral attention and limbic; FP = frontoparietal; DM = default mode network.

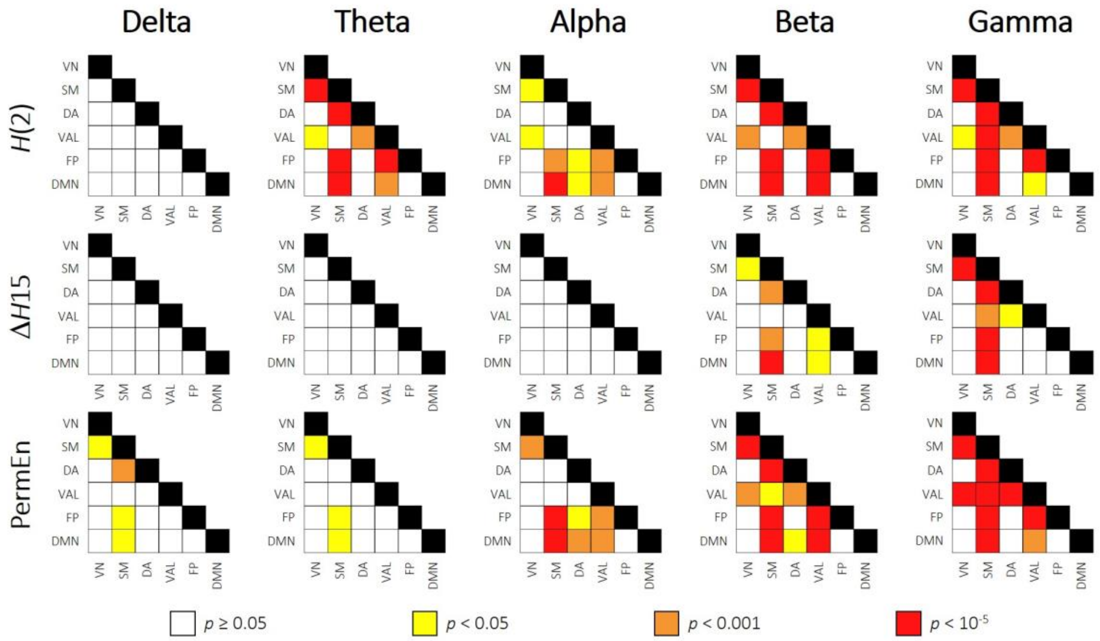

Figure S3. Pairwise comparisons of resting-state network dynamics based on 19 electrodes. The pairwise comparisons (using Bonferroni correction) verified the different nature of the SM and VAL networks, most strongly in the beta and gamma bands, regarding $H(2)$ and PermEn. Although there are subtle differences, in general these results are well in line with those acquired by analyzing data from all 62 channels. VN = visual network; SM = somatomotor; DA = dorsal attention; VAL = ventral attention and limbic; FP = frontoparietal; DM = default mode network.

		Delta	Theta	Alpha	Beta	Gamma
***H*(2)**	Friedman *p*	0.7524	**<0.0001**	**<0.0001**	**<0.0001**	**<0.0001**
	Kendall's *W*	0.0631	**0.2898**	**0.3321**	**0.5054**	**0.4897**
**Δ*H*15**	Friedman *p*	0.2786	0.5577	0.4848	**<0.0001**	**<0.0001**
	Kendall's *W*	0.0973	0.0764	0.0813	**0.2544**	**0.3138**
**PermEn**	Friedman *p*	**0.0042**	**0.0016**	**<0.0001**	**<0.0001**	**<0.0001**
	Kendall's *W*	**0.1748**	**0.1889**	**0.4151**	**0.6411**	**0.5961**

Table S1. Results of the Friedmann tests and Kendall's W coefficients using data from 19 channels. Except for Δ*H*15 in the delta band – where the Friedman test indicated significant spatial differences when all 62 channels were used – results were replicated almost identically, with most cases showing even higher Kendall's W values when using only 19 channels.
